# Evolving conservation: The role of unconventional approaches to restore contemporary vertebrate populations and genomic biodiversity

**DOI:** 10.1093/jhered/esag040

**Published:** 2026-05-21

**Authors:** Bridgett M vonHoldt, Matt James, Stephen J Gaughran, Alexandra L DeCandia, Klaus-Peter Koepfli, Dan Flores, Nucharin Songsasen, Kristin E Brzeski, Evelyn L Jensen

**Affiliations:** Department of Ecology & Evolutionary Biology, Princeton University, Princeton, NJ, United States; Colossal Biosciences, Austin, TX, United States; Department of Ecology & Evolutionary Biology, Princeton University, Princeton, NJ, United States; Department of Integrative Biology, University of California, Berkeley, Berkeley, CA, United States; Department of Biology, Georgetown University, Washington, DC, United States; Center for Conservation Genomics, Smithsonian’s National Zoo and Conservation Biology Institute, Washington, DC, United States; Colossal Biosciences, Austin, TX, United States; Department of History, University of Montana, Missoula, MT, United States; Center for Species Survival, Smithsonian’s National Zoo and Conservation Biology Institute, Front Royal, VA, United States; College of Forest Resources and Environmental Science, Michigan Technological University, Houghton, MI, United States; School of Natural and Environmental Sciences, Newcastle University, Newcastle Upon Tyne, United Kingdom

**Keywords:** back-breeding, conservation genomics, endangered species, extinct genomic variation, de-introgression, reviving

## Abstract

Conservation biology and restoration ecology are two essential yet distinct disciplines that address the growing challenge of biodiversity loss. Traditionally, these fields have relied on ecological principles and management practices aimed at protecting or reestablishing natural systems. The crisis is no longer just ecological; it is evolutionary and genomic. The accelerating pace of environmental change has outstripped the capacity of conventional approaches, creating a pressing need for innovative solutions. Biotechnology offers potentially transformative tools that can enhance the effectiveness and precision of both conservation and restoration efforts, especially for species where conventional conservation approaches have proved insufficient. Techniques such as genetic rescue, synthetic biology, and gene editing are increasingly being explored to address critical challenges, such as invasive species control, genetic diversity loss, and habitat fragmentation, to both invigorate endangered species and restore historical biodiversity. Despite its promise, the integration of biotechnology into conservation and restoration has raised ethical, ecological, and regulatory concerns. These include ecological unpredictability and public resistance to genetic interventions in wild populations. This perspective examines the current landscape of biotechnological applications in conservation and restoration, highlighting successful case studies, ongoing controversies, and optimism for additional progress. We argue that thoughtful, transparent integration of biotechnology that is grounded in ecological knowledge and stakeholder engagement can reconcile the goals of conservation and restoration. As ecosystems face mounting pressures, biotech-enabled strategies may prove essential for fostering resilience and ensuring long-term ecological sustainability.

## Tug of war between conservation and restoration

Conservation biology is a multidisciplinary and mission-oriented science focused on studying and protecting biodiversity. Decisions in conservation are shaped by value-based priorities that vary across communities and stakeholder groups, which can lead to heated debates of value systems and how actions are prioritized (Young 2000; Vaughn et al. 2010; Wiens and Hobbs 2015; Mori and Isbell 2024). With resources limited, species-focused conservation initiatives often struggle to move beyond preserving the current state of a species and preventing additional degradation. Successful programs tend to involve legislation to protect target populations and habitats through adjusted management practices. A conceptually different ambition in the field includes “biodiversity restoration,” which focuses on recreating a historical baseline by altering a contemporary unit (usually an ecosystem or habitat) to approximate a past ecological condition through reversing degradation and using inferential methods to fill in the missing components. In museum studies, “disruptive conservation” applies similar principles by restoring partial or damaged artifacts. The restored aspects may be viewed as inauthentic, but the restored object can now be viewed in its entirety, providing the viewer with authentic understanding (Sweetnam and Henderson 2022). Conservation biology has a long history of adopting a similar strategy in approaching species and ecosystem conservation, with ecologists and biologists determining which aspects are authentic and which gaps need to be filled.

A persistent source of confusion in conservation biology stems from the lack of clearly defined global goals and targets for biodiversity conservation, despite frequent references to preservation, restoration, and conservation as if they were independent or interchangeable efforts. In practice, these approaches reflect distinct values, objectives, and time horizons. A failure to articulate their differences undermines effective policy and implementation. Species-focused conservation and ecosystem restoration cannot proceed in isolation, as each depends on the other. Yet, their priorities and outcomes may diverge rapidly on evolutionary timescales when populations are already demographically depleted or genetically compromised. In such cases, ecosystem recovery alone may be insufficient to prevent extinction without direct species-level evolutionary and genomic intervention.

The Kunming-Montreal Global Biodiversity Framework (KM-GBF) represents the most recent international effort to reconcile these approaches by providing a roadmap for biodiversity protection while promoting sustainability. Adopted at the 2022 Conference of the Parties to the Convention on Biological Diversity, the KM-GBF establishes 23 urgent action targets organized around three broad objectives: 1) reducing threats to biodiversity, 2) meeting human needs through sustainable use and equitable benefit-sharing, and 3) developing the tools and solutions necessary to implement and mainstream these actions (CBD 2023). These action targets are slated to be achieved by 2030. Notably, one of the targets calls for halting species extinctions, safeguarding genetic diversity, and managing human–wildlife conflict. This objective implicitly requires active genetic and demographic management if natural recovery processes have been eroded. Achieving these goals will likely necessitate the judicious application of species-level interventions such as genetic rescue, managed gene flow (translocations), assisted reproduction, and emerging biotechnological approaches. Whether and how such tools are deployed must be guided by clear decision frameworks that integrate evolutionary risk, ecological context, and ethical considerations. This renewed global commitment must therefore succeed where previous efforts, including the 2020 Aichi Biodiversity Targets, have failed to halt extinctions. To achieve this, it is critical to recognize when conventional conservation measures have been insufficient and when targeted genetic and biotechnological interventions may become essential components of biodiversity recovery (Djoghlaf 2020).

Effective species conservation requires a clear decision-making framework that distinguishes when conventional approaches are sufficient from when more interventionist strategies become necessary. Conservation actions prioritize preventing further species decline with recovery plans that integrate habitat protection, reduction of anthropogenically-driven mortality, and maintenance of ecological connectivity to promote natural demographic and evolutionary processes. However, for a growing number of species, these approaches alone are not sufficient to support recovery, particularly when severe genetic diversity loss due to habitat fragmentation or population bottlenecks constrains recovery. In such cases, biotechnology represents a final tier of conservation action. Deployed judiciously and in conjunction with ecological and social safeguards, biotechnological approaches provide a means to recover lost genetic variation, restore demographic viability, or confer resilience that cannot be achieved through conventional methods alone. Framing biotechnology within this escalation pathway underscores its role not as a replacement for conservation, but as a necessary extension of it when all other options have been exhausted.

Today, the intersection of biotechnology and conservation evokes the hotly debated topic of de-extinction. As a relatively nascent science (Novak 2018), de-extinction’s practical utility and successful integration into conservation biology remain unproven. Although it represents a frontier application of biotechnology, it exists at the far end of a broad spectrum of possible interventions. Rather than wade into this divisive and yet-to-be-tested field, here we aim to highlight more immediate approaches that fall within the current scope of conservation practice.

## Restoration for a new future

Understanding the past is crucial to combating threats to our planet's future. Human activity in the past 50,000 yr has been a significant driver for the degradation of species diversity, species interactions, ecological functions, and natural evolutionary history (Sullivan et al. 2017). During the last several centuries, an epoch often referred to as the Anthropocene, the growing human population and expansion of industrial technology have accelerated the rate of global change through increased carbon emissions, habitat destruction and degradation, and direct overharvest of wildlife (Crutzen 2002; Waters et al. 2023; Edgeworth et al. 2024; Zalasiewicz et al. 2015, 2024). One downstream effect of this expanded anthropogenic impact is the rapid rate of current biodiversity loss when considering the background rate of extinction (0.1 extinctions per million species per year), which is 1,000-times higher than prehuman, deeper geological epochs (Dirzo et al. 2014; de Vos et al. 2015; Sullivan et al. 2017).

Between the start of the 16th century and 2025, the International Union for Conservation of Nature (IUCN) estimates that approximately 927 species have gone extinct, which include iconic species such as the Dodo bird in 1690 (*Raphus cucullatus*; Roberts and Olow 2003; Hume et al. 2004), the blue antelope (*Hippotragus leucophaeus*) in 1800 (Hempel et al. 2022), the quagga in 1883 (*Equus quagga quagga*; Heywood 2013), the passenger pigeon in 1914 (*Ectopistes migratorius*; Hung et al. 2014), the thylacine (Tasmanian tiger or wolf; *Thylacinus cynocephalus*) in 1936 (Carlson et al. 2018), the Caribbean monk seal in 1952 (*Neomonachus tropicalis*; King 1956), the baiji in 2002 (*Lipotes vexillifer*; Guo 2006), and the Pinta Island tortoise in 2012 (*Chelonoidis abingdoni*; Edwards et al. 2013).

Many additional extant species are threatened with extinction with a seemingly impossible road to recovery, as they face endless, near-insurmountable challenges of habitat loss, degradation, and fragmentation as the human-dominated footprint continues to encroach on the last natural ecosystems supporting wildlife (Liu et al. 2025). Humans convert habitats and biome distributions, whereas global climate change shifts seasonal phenology, desynchronizing major life-history events (e.g. leaf budding, egg laying, hibernation onset or emergence; Visser and Both 2005; Cohen et al. 2018; Inouye 2022). As a result, biodiversity is now impacted even in the world’s most remote pristine environments (Wolfe et al. 2025). Beyond outright extinction, many extant species now persist in a genetically impoverished state and have lost substantial standing genetic variation through population bottlenecks, fragmentation, and prolonged demographic decline. Such erosion of standing variation inevitably constrains adaptive potential and limits the capacity of an evolutionary response to emerging threats. Consequently, recovery efforts that focus solely on habitat protection or demographic growth may be insufficient unless they also seek to preserve or restore standing genetic variation. Those goals underpin long-term species viability.

## Conservation practices and partnerships are globally evolving

Conventional conservation practices typically encompass actions aimed at preserving habitats or components thereof, developing sustainable resource management, and implementing species-specific or ecosystem services-based conservation programs (Fetene et al. 2012; Robert et al. 2016). Many of the relevant tools include biobanking, breeding programs, and protected areas as part of a species survival program. We argue that the success rate of endangered species survival will be enhanced when new, unconventional biotechnological advances are paired with biological knowledge and traditional practices for ex situ conservation. These advancements can push conservation from merely preserving extant biodiversity to facilitating the restoration of lost species and landscapes, while simultaneously capturing the attention and imagination of the broader public. Pursuit of these goals requires innovative conservation partnerships that could include private (bio)technology companies and their nonprofit foundations, especially in times of dwindling financial support (Box 1). Examples include Colossal Biosciences/Colossal Foundation (https://colossalfoundation.org/), Revive & Restore (https://reviverestore.org/), Amazon.com/Bezos Earth Fund (https://www.bezosearthfund.org/), and Superorganism (https://www.superorganism.com/), to name a few. We are at a pivotal moment, as emerging private (bio)technology companies begin to play a role in species conservation, providing an opportunity to distribute the responsibility of protecting biodiversity across a broader coalition of stakeholders. Rather than conservation funding and action falling exclusively on government organizations and conservation NGOs, (bio)technology companies can help ameliorate the multifaceted hindrances to species conservation through their fundraising and innovation focus. Such collaboration has precedent: the original Human Genome Project was publicly funded, with the parallel emergence of several biotechnology companies in the private sector also sharing the common goal of precision medicine and healthcare (National Research Council 1988; US Congress 1988). Although the public effort experienced an increase in funding over the initial years of research and design, the biotechnology subsector received significantly higher financial investments (Cook-Deegan et al. 2001; Moses III et al. 2005; Wiechers et al. 2013). The outcome was a scientific race of innovation and discovery, moving technology leaps and bounds through collaboration and cooperation. Paired with conventional conservation practice, we believe biotechnology-based conservation provides additional opportunities to conserve and rebuild species biodiversity. Open-data mandates requiring the public release of data, technology transfer agreements providing royalty-free licenses for noncommercial conservation use, and dissemination across various media platforms can ensure that discoveries generated by (bio)technology companies reach and are shared with the broader conservation community. Moreover, adherence to the CARE Principles for Indigenous Data Governance (Carroll et al. 2020; Jennings et al. 2023) and the Access and Benefit-Sharing frameworks of the Nagoya Protocol (Secretariat of the Convention on Biological Diversity 2011) further ensures that advances respect the sovereignty of biodiversity-provider nations and local communities.

Box 1. The United States is falling behind

**Figure B1:**
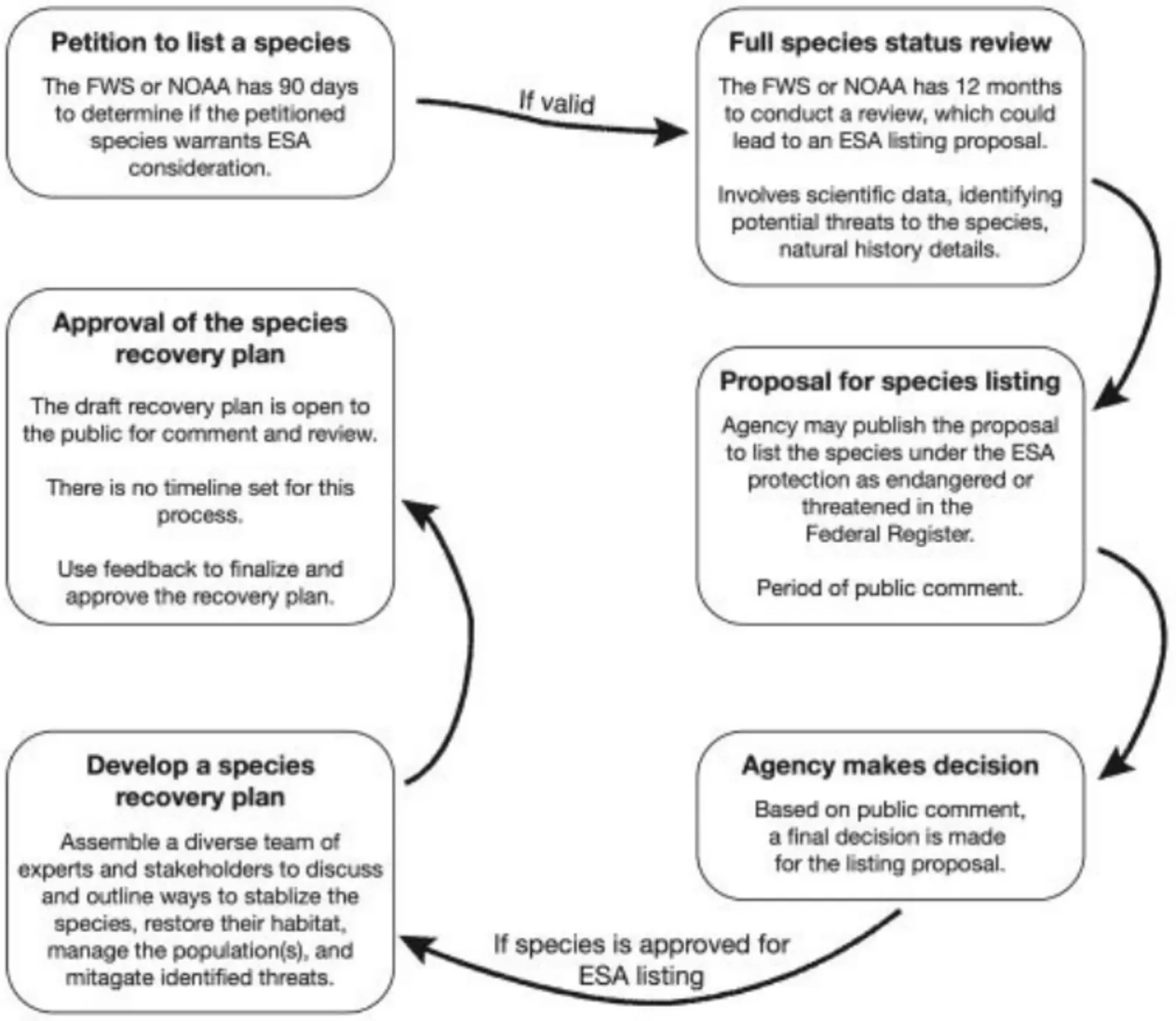
Flowchart of the bureaucratic process for developing and implementing an endangered species recovery plan.

The United States never officially approved the Convention on Biological Diversity agreement; thus, the nation does not adhere to the targets of the KM-GBF. Consequently, the United States continues to follow conventional methods and regulatory processes for endangered species protection and recovery. The general timeline from petitioning to list a species for protection under the Endangered Species Act (ESA), to implementing a ratified recovery plan is long and arduous, ranging from years to decades (Fig. B1). These recovery plans can incorporate both ex situ and in situ designs for restoring the species in the wild, although translocation of animals to ex situ environments is not common practice and usually only implemented as a final option to avoid extinction. We encourage the opportunistic collection and banking of genetic materials (e.g. viable reproductive cells and tissues) from in situ individuals, and the use of available technologies to augment genetic viability of ex situ populations. In the United States, species survival plans are implemented by zoos and other approved breeding facilities, with potential use at a later stage in the recovery plan for management of the wild population through reintroduction efforts or vice versa, where the ex situ population may be opened to add new founders for the long-term genetic viability of the breeding population. Regardless of the plan goals, success is only probable with full engagement of all stakeholder communities and extensive collaborations with conservation groups, scientists, policy makers, ethics and social scientists, landowners, local communities, and public educators.

The finances of species conservation are also troublesome. In the United States, the realized support for species recovery is low, likely due to several factors: ESA protections are provided when a species reaches critically low numbers and thus has a negative relationship to recovery time, there are few incentives for communities to participate in species recovery, and there is a substantial lack of funding (Wilcove et al. 1993; Brown and Shogren 1998; Schwartz 2008; Eberhard et al. 2022). A study of USFWS appropriations between 1992 and 2020 showed decades-long trends of funding deficits for imperiled species, limiting the capacity of USFWS to implement ESA actions (Eberhard et al. 2022). Increased funding would have a significant positive impact for conservation and avert species extinctions (Ferraro et al. 2007; Wilde 2014; Langpap et al. 2018). In 2023, the USFWS received a total annual budget of USD $3.7 billion (Supplementary Table S1), where roughly $1.2 billion was earmarked for imperiled species conservation. However, only a fraction of such species receives budgetary support (US FWS 2023). Two fish species (salmon and steelhead trout) enjoy about half of the annual budget allocation, with 7% for mammals, 5% for birds, 2% for plants, and <1% to insects. The majority of imperiled species receive <$1,000 toward their annual recovery efforts. When species conservation is found under the purview of big international nongovernmental organizations, itemized budgets vary. For example, in 2024, the World Wildlife Foundation deployed $402 million in program spending, which included $32 million for wildlife, $26 million for forest, and $14 million for ocean conservation and policy programs (WWF 2024). Conservation International spent $132.1 million on field-based conservation programs (Conservation International 2023), and the Association of Zoos & Aquariums reports an average annual spending budget of $160 million on conservation initiatives (AZA 2024). The Cooperative Endangered Species Conservation Fund is a federal financial assistance program available to support listed, at-risk, and candidate species on nonfederal land (https://www.fws.gov/program/cooperative-endangered-species-conservation-fund).

## Approaches for unconventional demographic and genetic restoration

Conservation biology has a long history of combining conventional management strategies with technological advances. For example, molecular genetics and genomics have been successfully integrated into management plans for decades, as has the building of tissue biobanks as living archives of biodiversity, such as the Frozen Zoo concept (Benirschke 1984; Soulé et al. 1985; Bolton et al. 2022; Mooney et al. 2023). These banks are composed of cryopreserved viable cells, tissues, gametes, or embryos, even without the explicit biotechnology yet available for effective use of such precious biomaterials. These living banks safeguard a snapshot of genomic and taxonomic diversity, offering a tangible opportunity to revive and enhance imperiled species. They achieve this through ex situ (in facilities under human care) breeding programs, specialized genetic and disease research, and by serving as a vital link to in situ (wild or natural) populations.

To logically escalate the conservation pathway, we structure our argument as a tiered decision framework that begins with conventional conservation, next integrating genetic management, and finally building and deploying biotechnological tools. Biotechnology has expanded the realized value of these banked biodiversity resources with key methodologies that are now real options for the demographic (i.e. increasing reproduction and abundance) and genetic restoration of endangered species. It is the very existence of these banks that enables the innovation of some types of biotechnology interventions, whereas others harness genetic resources found within living organisms, such as utilizing admixed individuals or related species as templates for gene editing. Here, we highlight some of the biotechnology solutions that work to restore, preserve, and actively rebuild lost genetic variation.

### Extensions of conventional practice

Gamete cryopreservation and Assisted Reproductive Technologies, which include artificial insemination (AI) and stem cell technologies, as well as in vitro embryo production and transfer, have played critical roles in maintaining and restoring genetic diversity in ex situ wildlife populations (Korody et al. 2021; Hutchinson et al. 2024). An excellent example of a species that benefited from the advancement of these technologies is the black-footed ferret (*Mustela nigripes*), the only member of the genus *Mustela* endemic to North America. Once numbering in the hundreds of thousands, the species suffered a severe population decline driven by the eradication of its primary prey, prairie dogs (*Cynomys* spp.), and its high susceptibility to diseases such as canine distemper virus and sylvatic plague, leading to its presumed extinction in 1979. Following its rediscovery in 1981, an ex situ breeding colony was successfully established in 1986, which eventually became the source for restoring the species across its former expansive range (Anderson et al. 1986; Roelle et al. 2006; US FWS 2013). Since the first successful reproduction and weaning in 1987, an assumed seven genetic founders have led to the birth of well over 11,000 kits. Roughly 650 animals are currently living ex situ under human care and in situ at a handful of reintroduction sites (Marinari and Lynch 2023). Given the severity of their population bottleneck, black-footed ferrets exhibit limited genetic diversity (Wisely et al. 2002, 2008). As such, genetic management of this endangered species has been a high priority since the Black-Footed Ferret Recovery Plan was founded.

### Recovery of lost genetic diversity

To minimize genetic diversity loss within ex situ populations, traditional approaches to genetic management include calculation of mean kinship from the pedigreed studbook and collaborative management across multiple ex situ facilities to recommend pairings (Roelle et al. 2006; US FWS 2013). These pairings often occur naturally but are also facilitated by AI from fresh or cryopreserved sperm, when needed (Howard et al. 2016a). To date, approximately 140 individual black-footed ferrets have been born by AI, including kits produced from sperm that had been frozen for 10 to 20 yr (Howard et al. 2016a). Although AI with cryopreserved sperm of founders with low mean kinship can improve genetic diversity by 0.2% (Howard et al. 2016b), this technology relies only on male gametes and on the successful collection of semen samples from all individuals. After decades of captive breeding, low founder numbers, and a high mean kinship of the ex situ black-footed ferret population, conventional conservation has failed to restore sufficient genetic diversity (Novak et al. 2024). To ensure that the genetics of all individuals are represented in the contemporary population, the Black-Footed Ferret Recovery Program has recently adopted advanced biotechnological approaches for conservation. This includes successful conservation cloning of cryopreserved cells from a female that died in 1988 and left no living descendants in the current population (Novak et al. 2024). Through somatic cell nuclear transfer (SCNT), three healthy black-footed ferret kits named Elizabeth Ann, Noreen, and Antonia were produced from this new, eighth founder Willa (studbook #10) of the ex situ population. Critically, one of these cloned kits (Antonia) successfully gave birth in June 2024, thereby contributing her resurrected genetic material to the black-footed ferret population. This advancement moves beyond preservation of genetic diversity to facilitate the true restoration of variants that were previously lost in this species. Cell lines were generated and cryopreserved from two individual black-footed ferrets, Willa and a male (studbook #2), to aid in the restoration and improvement of genetic diversity. SCNT using immortalized cell lines nonetheless provides a powerful tool for genetic rescue when integrated with comprehensive conservation breeding programs, and the successful cloning demonstrated in black-footed ferrets as well as Przewalski’s horses (*Equus przewalskii*) from cryopreserved cells collected in 1980 (Novak et al. 2025) strongly supports expanding efforts to generate and biobank cell cultures from current extant populations of threatened species (Ryder and Onuma 2018).

### Directed genomic change

Some of the most intractable conservation challenges may now become feasible with genome editing and gene drive technologies. The latter, made possible by recently emerged gene editing technologies like CRISPR/Cas9, can be used to create targeted nucleotide changes in wild organisms that circumvent the expected rules of inheritance (probability of inheritance >50%), rapidly spreading new edited allele(s) throughout the population via sexual reproduction (Dance 2015; Esvelt and Gemmell 2017; Piaggio et al. 2017; Hartley et al. 2022). The applications vary from manufacturing and driving a genetic variant that can hinder the reproduction of invasive species (Teem et al. 2020) to pathogen resistance in endangered species (Yin et al. 2024). Collectively referred to as “synthetically-assisted conservation” (Brodie et al. 2025), these unconventional methods to modify or introduce new genetic variants designed to change organismal fitness have been around for decades, with new envisioned applications for combatting disease, climate change, and competition from invasive species, or assisting with the de-extinction of species (Esvelt and Gemmell 2017; Novak 2018; Fidelman et al. 2019; Godwin et al. 2019; Reynolds 2021). The IUCN recently adopted the world’s first policy on synthetic biology for the conservation of nature, a statement that provides hope for imperiled species while also ensuring that biotechnology applications proceed with caution as new efforts include “other sectors” to complement ongoing conservation efforts (IUCN 2025).

One prominent example of the application of synthetic biology involves the cane toad, aimed at mitigating the species’ devastating impact on native fauna. This invasive species in Australia was originally introduced as a hopeful means of controlling an agricultural insect pest but subsequently devastated much of the nation’s predators for the past century because of the bufotoxin it secretes from its salivary glands (Lever 2001; Phillips et al. 2010; Shine 2010; Jolly et al. 2015). Among the species most impacted is the endangered northern quoll, where populations are significantly dwindling due to predation (Hill and Ward 2010) and cane toad bufotoxin (Ujvari et al. 2013). Some predatory mammalian species coevolving with bufotoxin-secreting species have developed molecular adaptations, specifically an amino acid change in the *ATP1A1* gene that grants them resistance to the toxin (Ujvari et al. 2015). This provides a specific target for gene editing and drive in an endangered species that otherwise could not acquire the adaptations from an evolutionary response in time to prevent extinction (Ibri et al. 2024). Gene drives have already been successfully implemented in several insect species, predominantly with the anticipated deployment for disease and pest control (Gantz and Bier 2015; Gantz et al. 2015; Hammond et al. 2015; Gurr and You 2016; Medina 2017; Scott et al. 2017; Wedell et al. 2019; Li et al. 2020).

### Reconstruction of extinct genomes and restoration of ancestral genomic variation

Ancestral genetic variation from endangered or extinct species can persist in the admixed genomes of their closely related contemporary extant species (Carlson et al. 2014). Developing back-breeding programs explicitly designed for human-directed hybridization can result in the de-introgression or recovery of parental genomes that are either at risk of extinction or are extinct. Practically, de-introgression programs prioritize animals that carry targeted genomic material (e.g. variants from the extinct species), as determined from genomic analysis, for use in a breeding program designed to concentrate the targeted variation that resides as fragments in an admixed population (Shapiro 2017; Forshaw et al. 2025). By bringing these formerly disparate chromosomal fragments together into the same genome, one can essentially rebuild the genomic composition and diversity of the extinct species. This strategy has been successfully applied to restore the genomic variation of an extinct bovid, aurochs (Loftus et al. 1994; Edwards et al. 2007; Stokstad 2015), and has been proposed for the Scottish wildcat (Lawson et al. 2025). The focus of de-introgression is similar to standard ex situ breeding programs: minimize kinship, maximize heterozygosity and genomic uniqueness, while further minimizing off-target genomic ancestry (Lawson et al. 2025). The specifics of a de-introgression breeding program require real-time genomic monitoring for local and global ancestry assessment, with significant scrutiny during the foundation of the initial pedigree for the breeding population (Lawson et al. 2025).

A challenge for such programmatic design is when ecosystem restoration relies on restoring species that are long-lived, such as the tortoise species of Floreana Island (Box 2). In lieu of a back-breeding program to reconstruct the purest approximation of the extinct Floreana Island tortoise (*Chelonoidis niger*), the program values admixed genomes to boost genomic diversity that should aid adaptive evolution and resilience (Miller et al. 2017; Cayot and Hunter 2021). By embracing the principle that an “imperfect” population is preferable to extinction, such a viability-centered restoration approach establishes a precedent for biotechnologically assisted recovery efforts in other species, while explicitly acknowledging trade-offs in ancestry and genomic composition.

Box 2. The extinct Floreana giant tortoise lineage

**Figure B2:**
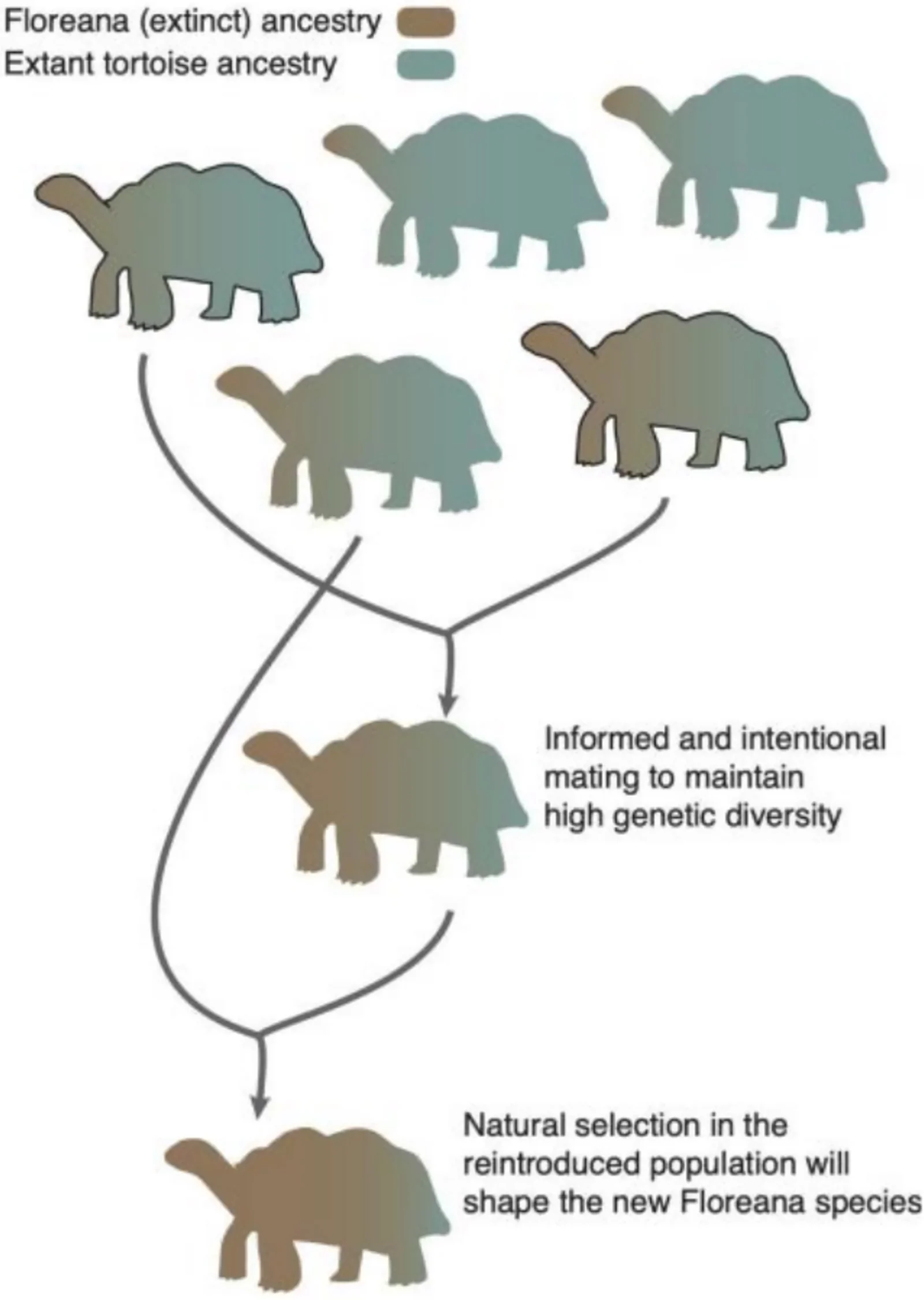
Schematic of intentional breeding to enrich and maintain high genomic diversity.

Hybrids and complex admixtures are typically not assigned much conservation value or protection, especially in cases where the interbreeding arose through human actions (Jackiw et al. 2015), which has implications for the potential future status of species restored through certain types of biotechnology. The Floreana Island giant tortoise (*Chelonoidis niger*) is an example of a pragmatic approach to species and ecosystem restoration using species admixtures. One of the 15 lineages of Galapagos giant tortoise, the Floreana tortoise was driven to extinction through overexploitation around 1840. However, it lives on in the form of complex species admixtures. Sailors inadvertently translocated tortoises to Isabela Island, where they subsequently interbred with the local tortoise (*Chelonoidis becki*). When genetic analyses first identified signatures of this interbreeding (Poulakakis et al. 2008), there was immediate interest in establishing a captive breeding program to restore tortoises to Floreana Island with ancestry from the original lineage (Quinzin et al. 2019; Hennessy and Gibbs 2022). Back-breeding to increase *C. niger* ancestry is conceptually appealing; however, the logistics of breeding multiple generations of such long-lived organisms are impractical given their long generation time combined with an urgent need for tortoises to resume their ecosystem services to restore Floreana Island. Therefore, rather than aiming to breed the purest approximation of *C. niger* through backcrossing, the objective of the ongoing captive breeding program is to maintain high levels of genetic diversity (Miller et al. 2017) (Fig. B2). The Galapagos National Park has taken a long-term view that natural selection will allow the population to evolve adaptations to Floreana Island, with the expectation that the heightened levels of genomic variation found in admixed genomes may enhance the population’s resilience (Cayot and Hunter 2021). In February 2026, more than 150 captive-bred mixed ancestry tortoises were released on Floreana Island to resume the ecological role of their ancestors that had been unfilled for almost two centuries. This pragmatic approach to restoration—that it is better to have an “imperfect” population than none at all—could benefit other species where biotechnology interventions could assist recovery, but at the expense of lineage purity.

There is an effort underway to utilize this strategy to reintroduce variants from the ancestral Red Wolf (*Canis rufus*) genome that were lost during the species’ severe decline that resulted in it being declared extinct in the wild in 1980 (US FWS 1989). Coyotes currently living in the American Gulf Coast states of Texas and Louisiana are known to carry substantial amounts of ghost Red Wolf ancestry, possibly representing extinct Red Wolf genomic variation that has been naturally maintained (Heppenheimer et al. 2018, 2020; vonHoldt et al. 2022a, 2022b; vonHoldt et al. 2026). An ex situ back-breeding program would significantly increase the knowledge for conservation practitioners to understand the potential success of concentrating ancestral Red Wolf chromosomal ancestry blocks into the genomes of a few, unrelated individuals, which could serve as a source for genetic rescue for the critically endangered Red Wolf. The genomic tools already exist to monitor chromosomal ancestry blocks that are unique to preextinction Red Wolves, in addition to the current ex situ Red Wolf breeding population (vonHoldt et al. 2022a, 2022b, and unpublished data). Thus, a human-directed breeding program could be highly successful in reviving previously extinct genomic variation and restoring this variation to the ecosystems once occupied by the Red Wolf. Paired with wild-fostering or translocation, back-breeding provides a foundation for integrated (i.e. inter situ) conservation strategies; these allow natural processes to drive the restoration of the species' ecological function. Several of the coauthors of this essay have received financial support for a pilot study to test such a program, which is underway. Such an initiative was discussed for several years among the relevant conservation stakeholders but was finally catalyzed by the discovery of the ghost ancestry within the Red Wolf genome.

## Life after biotechnology approaches

Human diversity and experience shape personal and professional value systems. As such, it is unlikely a unanimous univocal view will emerge with respect to the outcomes of the applications of biotechnology to conservation, but thoughtful discussions of these strategies, their consequences, and the consequences of inaction will help maximize successes. Existing species concepts are not equipped for the outcomes of conservation biotechnology, a challenge that becomes especially clear when species regulatory systems rely on static taxonomy. Here, we briefly address a few topics anticipated to arise when biotechnology efforts are used for genetic and demographic restoration.

### What do we call them?

The ethical and technical underpinnings for species revivals and restoration often face a debate about “authenticity” values, justification, rationale, and hubris (Sandler 2013; Novak 2018), with some voicing concerns regarding static species concepts (Patten 2015; Slater and Clatterbuck 2018; Powell 2023) or that our current catalog of species concepts does not accommodate organisms that are the outcome of biotechnology interventions, gene editing, or adjusted genomic composition through back-breeding and de-introgression. The outcomes of biotechnologies to restore demography and genomic diversity essentially skip the phylogenetic evolutionary processes of natural selection, hybridization and lineage sorting, coevolution, and adaptations. Thus, species that have been “tampered with” are not well accommodated within the current collection of “authentic” species concepts. The field of evolutionary biology has debated species concepts and their universal applicability for decades, with progress in some areas but little agreement in others (Stankowski and Ravinet 2021). This issue is not just academic, because taxonomic boundaries are the basis of regulatory frameworks for species protection. For species to benefit from restoration through biotechnology, our field must evolve language and develop our species concepts to accommodate biological processes such as genetic modifications via back-breeding, gene drives, or cloning of entirely new genetic variation, which currently does not exist within the species’ description. The unified species concept (de Queiroz 2005, 2007) may provide a good starting place for this. The central pillar of this species concept is that a species is a “separately evolving metapopulation lineage,” with individual properties (e.g. morphology, monophyly, reproductive isolation) providing support for the species assessment. If the outputs of biotechnology are integrated into existing populations or form new separately evolving metapopulation lineages, they may be recognized as species under the unified species concept regardless of their evolutionary origins. We argue that conservation practitioners and regulatory policies should move toward process-based definitions of species and evaluate interventions based on the species function, lineage continuity, and restoration outcomes.

### Where do we rewild them?

Ecosystems are complex, and research is only starting to understand how they assemble, evolve, reorganize, collapse, and maybe recover (Forman 1995; Whitham et al. 2006; Bascompte and Stouffer 2009; Leibold et al. 2022; Keith et al. 2023). The species selected for biotechnological interventions are based on ecosystem restoration goals, surrogate and model species availability, and those most at risk of extinction. At the core of restoration is the focus to rebuild the components for the needed and missing ecological functions within an ecosystem. Biotechnologies can assist in the production of individuals that fulfill these functions through cloning, gene editing, or back-breeding. Similar to all new biomedical treatments and options, biotechnology needs time to develop, assess, evaluate, and pilot new options. The conservation community is entering a new era where pilot studies are needed to determine the success of a biotechnological approach well before the critical moment of deployment is on us. The likely first steps for biotechnology outputs will be animals living under human care in an ex situ environment, which results in improved demographic vital rates such as survival, recruitment, and population growth. However, the conventional conservation efforts of community engagement, habitat and species assessments, and developing recovery plans need to be matured and prepared for incorporating biotechnology. This process requires that breeding facilities, reintroduction sites, and community support frameworks clear significant federal regulatory hurdles and establish the necessary infrastructure before the final step of rewilding begins.

### What are the fitness impacts?

As the Anthropocene is defined as a human-modified world, restored species are likely to find themselves entering novel, perhaps unrecognizable, environments and climates. The immediate selective pressures imposed by anthropogenic environments will favor traits that promote tolerance of human activity, infrastructure, chemical exposure, and the shifting realities of climate change (Palumbi 2001; Hendry et al. 2008; Darimont et al. 2009; Whitehead et al. 2012; Otto 2018; Wood et al. 2021). Speciation may accelerate within these modified landscapes as isolated populations adapt to divergent selective pressures, or the opposite may result—a full collapse of the restored species (Ålund et al. 2025). The increasing impact of deleterious alleles is predominantly attributed to the decreasing population size concomitant with increasing genetic isolation, leading to inbreeding and genomic degradation that are amplified by detrimental fitness impact, resulting in a reduction in species persistence (Kyriazis et al. 2021; Robinson et al. 2023). Biotechnologies can be thus leveraged to help minimize the impact of deleterious alleles on a restored species via significant genome screens and prediction models Ng and Henikoff 2001; Allen et al. 2019; Kang et al. 2020; Liu et al. 2022; Zhang and Jiang 2022). The predicted role of deleterious alleles in a genetically restored species is largely dependent on the species’ demographic history and thus needs to be supported by a robust a priori genomic surveillance and foundational knowledge of demography, ecology, and biology (Fitzpatrick and Funk 2019; Wilder et al. 2020, 2023; Kyriazis et al. 2021; Robinson et al. 2023; Kardos et al. 2025) (Box 3). Simulation approaches for genomic forecasting offer a powerful means of predicting fitness benefits and costs (Lotterhos 2024; Fitzpatrick et al. 2026). These tools allow genetic insights to be integrated into recovery plans tailored to a species' specific history—whether they have faced recent severe bottlenecks or have persisted as small populations over long evolutionary timescales. Additional advances in genomic predictive models combined with phenotypic studies and large sample sizes of allelic effects will allow for the identification of deleterious alleles, which could then be potentially removed or repaired from the population using gene editing technologies.

## Fundamental requirements of genetic and demographic restoration

Biotechnological options and their success depend on foundational ecological and evolutionary knowledge. To effectively apply biotechnology to species conservation, a thorough understanding of biology, ecology, and natural history must be the highest priority. However, the biology of fewer than 10% of extant species has been described, and studies have revealed a vast diversity in reproductive mechanisms—even among species within the same taxa (Mastromonaco and Songsasen 2020). The use of model species to generate basic biological knowledge has driven the development and application of biotechnological tools designed to restore the demographic and genetic diversity of threatened species, such as the black-footed ferret (e.g. domestic ferrets carried and birthed the black-footed ferret clones). However, challenges persist, particularly for novel or unique species that lack a closely related model species, which in turn requires thorough studies for each individual species. This requirement slows the progress of biotechnology applications in species conservation.

Box 3. Predicting genetic modification impacts on fitness and demography

The rate at which climates and habitats are shifting, adaptive evolution lags in providing the advantages species need at the time of need (Quintero and Wiens 2013; Cornford et al. 2023). Endangered species face a triple threat of reduced abundance and inbreeding in a quickly changing world, with fewer meiotic opportunities occurring to provide new advantageous alleles that could respond to these pressures. The emergence of biotechnology for genetic and demographic restoration provides new approaches, like gene editing and gene drives, by which a species-specific restoration program might benefit. Even with targeted removal of deleterious alleles known to impose fitness costs, or the addition of alleles conferring resistance to specific pathogens or diseases, our ability to predict the consequences of such edits under future environmental pressures is likely to be limited.

*Rapid response through gene editing.* When an endangered species faces specific threats, the primary goal of conventional conservation is to provide triage for the species while mitigating the threat. The diversity of threats can be deeply complex, historically or culturally ingrained, and expansive with roots in anthropocentrism (Mylius 2018). Those challenges must always be addressed for species restoration to be successful in the long-term. However, the need to assist endangered species is urgent; removing an anthropocentric threat requires more time than what many endangered species have. Threats from disease, pathogens, toxins, pollutants, and invasive/introduced species can be potentially ameliorated with biotechnology (compared with biological evolution) when a significant foundation of knowledge regarding their source and impact on the species is already available. Gene editing is a promising approach to eliminate inherited diseases, improve immunosurveillance, and remove pathogen, toxin, and pollutant vulnerabilities by providing immediate tolerance, resilience, or even resistance. Gene edits that are driven in an invasive, pest, or introduced species can hinder reproduction with the dissemination plan and degree of drive tailored to the species demography and life-history characteristics.

*Pleiotropy and other unintended outcomes.* When genes are edited using the CRISPR-Cas system, the intended results are screened for unintended, off-target consequences through a labor-intensive assessment of genomic data (Harrison and Hart 2017; Doench 2018; Pickar-Oliver and Gersbach 2019; Hanna and Doench 2020; Replogle et al. 2022; Ang et al. 2023; Mackay and Anholt 2024). Such consequences are often the result of breaking the double-stranded DNA molecule to make the intended changes (e.g. remove, replace, or repair some nucleotide sequence). Specific guide RNAs and other targeting molecules are designed or selected to make the intended edits, followed up with large-scale screens of the edited genomes for efficacy and environmental effects.

Genes often participate in shared developmental, metabolic, or regulatory pathways, such that modifying one component alters downstream traits in unanticipated ways. Even when off-target mutations can be minimized, edits to functional loci can generate pleiotropic effects, whereby a single genetic change could influence multiple phenotypic traits (Mackay and Anholt 2024). Such pleiotropy can create evolutionary trade-offs. For example, improving one adaptive trait such as pathogen resistance could simultaneously compromise another (e.g. body size, growth rate, reproductive output, or behavior). Postediting screens could suffer from limited assessment to only a subset of traits or environmental conditions. As a result, pleiotropic trade-offs may only become apparent after edited individuals are produced and evaluated across life stages or ecological contexts.

Additional concerns develop only after the edits have been selected and successfully implemented, producing a viable animal that is hoped to thrive. The long-term effects of gene editing are not fully understood on both the organism itself and how it functions in ecosystems. The editing process may be successful, but concerns can still present if the edits cannot function properly (or become irrelevant) for the animal in its natural habitat. Given the pace of environmental changes, the edits made and released now into an ecosystem could also present yet another mismatch in the near future due to human-derived modifications or climate change.

*There is no time to wait.* The benefits currently outweigh the risks, but such actions are not taken in a vacuum regarding the ethical considerations on both sides of the debate: To what end do we design and transfer adaptations? Will such triage ignite a deeper shift away from anthropocentrism and toward an environment-focused value system? Our planet is facing the sixth mass extinction event (Ceballos et al. 2015). Biodiversity is tied directly to essential life-sustaining factors like crop pollination, water purification, and global temperatures. The downstream effects of reduced biodiversity include significant structural challenges that impact humanity’s potential to feed ourselves, heal ourselves, and support future generations. To make it happen, the conservation community needs to increase its appetite for risk. Technological innovations are vital to this fight, which can spur even more innovation. We need to incentivize positive changes across industries by funding conservation, not just the reactive models employed for decades, but the proactive programs that are harnessing science and technology to our collective benefit.

For example, although SCNT holds immense potential for genetic and demographic restoration, several biological factors still influence the success of this technology in producing live offspring. Cloned kittens of the African wildcat (*Felis lybica*), sand cat (*Felis margarita*), and caracal (*Caracal caracal*) have been successfully generated through SCNT after their embryos were transferred into domestic cat recipients. However, this technique has yet to yield live offspring in other wild felid species, such as the black-footed cat (*Felis nigripes*), rusty-spotted cat (*Prionailurus rubiginosus*), the endangered flat-headed cat (*Prionailurus planiceps*), and the marbled cat (*Pardofelis marmorata*) (Gómez et al. 2006). This is likely due to a combination of incomplete epigenetic reprograming of the differentiated nucleus and incompatibilities between the donor cell and recipient cytoplasm related to mitochondrial heteroplasmy. Therefore, additional research is essential to better understand the epigenetic barriers associated with SCNT-mediated reprograming, ultimately increasing its efficiency and expanding its application across a broader range of species within the same taxa (Zhang et al. 2021).

Beyond reproductive technologies, the application of conservation biotechnology more broadly requires detailed knowledge of species-specific genomic architecture, life history, population structure, and selective environments. For interventions such as genetic rescue, backcrossing or de-introgression, assisted gene flow, or genome editing, it is essential to understand baseline levels of standing genetic variation, the distribution of putatively deleterious versus adaptive alleles, linkage relationships among loci, and how these variants interact with social structure and demography. Without this foundational knowledge, biotechnological interventions risk introducing alleles that have context-dependent effects, disrupt locally-adapted gene complexes, or provide short-term gains that are not sustained under future environmental or ecological pressures. Importantly, many of these parameters cannot be inferred solely from extant populations, particularly for species that have experienced severe bottlenecks, admixture, or long-term anthropogenic pressure. Computational simulations using forward-in-time population genetic models therefore represent a critical prerequisite for evaluating the potential benefits and limitations of biotechnology-based interventions before they are implemented in the wild. Such frameworks can quantify how many alleles or haplotypes might realistically be reintroduced, how quickly drift may lose them, and whether their retention meaningfully improves effective population size, adaptive potential, or long-term viability under plausible management and environmental scenarios. These approaches allow conservation practitioners to test alternative strategies, identify diminishing returns, and explicitly incorporate uncertainty into decision-making.

In the case example of the Red Wolf, simulations can be used to evaluate how much historical genetic variation could plausibly be recovered through back-breeding or de-introgression from admixed Gulf Coast canids, given known patterns of ancestry, linkage, and demographic history. These analyses can estimate the expected number and genomic distribution of Red Wolf-derived (extinct or extant) variants that could be reintroduced, the number of generations required for their retention, and the extent to which such variation would increase representation and resilience. Critically, simulation-based evaluation helps distinguish between theoretical potential and biologically realistic outcomes, ensuring that biotechnology is applied as a complement to ongoing conservation efforts.

## Reasons for grief and hope

As conservation biologists, we live in a world of constant loss, grief, or mourning (Hobbs 2013), experienced through species extinction, habitat degradation, or simply change over time. We use the scientific method to study what was lost and the aspects of how such loss occurred. We use the knowledge gained from analyzing past data in hopes of building accurate predictive models to help identify high risk situations for immediate action to halt future biodiversity losses. In some cases, we face the difficult situation where loss cannot be reversed, and extinction is imminent. Anger is a rising emotion, often accompanied by rejection when offered alternative, new strategies that deviate from conventional approaches. Conservation biology is facing its own crisis, with internal conflict regarding when, if, and how biotechnology can join the fight against loss (e.g. Seddon 2017). Conservation efforts must evolve from a focus on preservation to a future of reconstruction and restoration. Protecting what remains is no longer enough; we must actively rebuild genetic and ecological capacity. Genetic diversity is the limiting currency of species recovery. Biotechnology offers tools capable of recovering lost genetic diversity, or conferring resilience where natural processes and traditional management cannot operate on ecologically relevant timescales. Biotechnology is not optional and in fact is inevitable for some species when used as a last-tier, evidence-based tool. When applied transparently, under rigorous scientific and regulatory oversight, and when conventional options have been exhausted, biotechnological interventions represent a responsible extension of conservation practice rather than a departure from it. As conservation biology confronts a future defined by irreversible change, the field must diversify its toolkit to match the unprecedented scale of the crisis. Refusing biotechnology when extinction is otherwise inevitable risks substituting philosophical caution for biological loss. The decision is no longer whether intervention is desirable, but whether inaction is acceptable.

## Supplementary Material

Supplemental_Table_S1_esag040
