# Supplementary material for: Evolving conservation: The role of unconventional approaches to restore contemporary vertebrate populations and genomic biodiversity
Source: J Hered. 2026 May 21;117(5):993–1007. doi: 10.1093/jhered/esag040 (PMC13539457; doi:10.1093/jhered/esag040)
Supplement: Supplementary file 1 [file supplemental_table_s1_esag040.docx]

**Supplemental Table S1.** The 2023 summary of FWS’s appropriations (US FWS 2023).

| Item | Budget $ USD | Mission |
| --- | --- | --- |
| Ecological Services | 152.8 million | Clean energy solutions |
|  | 54.3 million | Conservation of at-risk species |
|  | 125.1 million | Species recovery |
|  | 23.9 million | Completion of 5-year species status reviews |
| Partners for Fish and Wildlife Program | 67.4 million |  |
| Coastal Program | 16 million |  |
| National Wildlife Refuge System | 426.6 million | Operations of wildlife and habitat management, visitor services, law enforcement, planning |
|  | 171.3 million | Refuge maintenance |
| Migratory Bird Management Activity | 70.2 million | Modernize permitting process |
| Law enforcement programs | 99.3 million | Investigates wildlife crimes and enforces laws that govern wildlife trade |
| International Affairs | 31.8 million | Granting program to assist other countries’ conservation and climate change efforts |
| National Fish Hatcheries | 260.4 million | Conservation, restoration, hatchery maintenance and operations, invasive species control |
| Cooperative Landscape Conservation Program | 19 million | Develop landscape conservation objectives to promote biodiversity |
| Science Support | 38.5 million | Adaptive science work to design and implement conservation and habitat management strategies |
| General Operations | 188.5 million |  |
| Construction | 46.4 million | Modernize communication tools |
| Cooperative Endangered Species Conservation Fund | 23.7 million | Supports state and local efforts to recover listed species |
| North American Wetlands Conservation Fund | 46.5 million | Wetland and waterfowl conservation |
| Multinational Species Conservation Fund | 19 million | African and Asian elephants, rhinoceroses, tigers, great apes, marine turtles |
| Neotropical Migratory Bird Conservation Fund | 7.9 million |  |
| State and Tribal Wildlife Grant | 82.4 million | State management of non-game species |
| Fixed-cost increases | 39.5 million |  |
